# Clinical and genetic factors associated with warfarin maintenance dose in northern Chinese patients with mechanical heart valve replacement

**DOI:** 10.1097/MD.0000000000005658

**Published:** 2017-01-13

**Authors:** Rui Liu, Jian Cao, Qian Zhang, Xin-Miao Shi, Xiao-Dong Pan, Ran Dong

**Affiliations:** a Department of Cardiac Surgery, Beijing Institute of Heart, Lung and Blood Vessel Disease, The Key Laboratory of Remodeling-related Cardiovascular Diseases, Beijing Anzhen Hospital, Capital Medical University, Beijing; b Department of Pediatrics, Peking University First Hospital, Beijing; c Department of Epidemiology, Beijing Institute of Heart, Lung and Blood Vessel Disease; d Experimental Center, Beijing Institute of Heart, Lung and Blood Vessel Disease, The Key Laboratory of Remodeling-related Cardiovascular Diseases, Beijing Anzhen Hospital, Capital Medical University, Beijing, China.

**Keywords:** dosing algorithm, mechanical heart valve replacement, northern Chinese, pharmacogenetics, warfarin

## Abstract

The effects of genetic variants on warfarin dosing vary among different ethnic groups, especially in the Chinese population. The objective of this study was to recruit patients through a rigorous experimental design and to perform a comprehensive screen to identify gene polymorphisms that may influence warfarin dosing in northern Han Chinese patients with mechanical heart valve replacement. Consenting patients (n = 183) with a stable warfarin dose were included in this study. Ninety-six single nucleotide polymorphisms (SNPs) in 30 genes involved in warfarin pharmacological pathways were genotyped using the Illumina SNP GoldenGate Assay, and their associations with warfarin dosing were assessed using univariate regression analysis with post hoc comparison using least significant difference analysis. Multiple linear regression was performed by incorporating patients’ clinical and genetic data to create a new algorithm for warfarin dosing. From the 96 SNPs analyzed, *VKORC1* rs9923231, *CYP1A2* rs2069514, *CYP3A4* rs28371759, and *APOE* rs7412 were associated with higher average warfarin maintenance doses, whereas *CYP2C9* rs1057910, *EPHX1* rs2260863, and *CYP4F2* rs2189784 were associated with lower warfarin doses (*P* < 0.05). Multiple linear regression analysis could estimate 44.4% of warfarin dose variability consisting of, in decreasing order, *VKORC1* rs9923231 (14.2%), *CYP2C9∗3* (9.6%), body surface area (6.7%), *CYP1A2* rs2069514 (3.7%), age (2.7%), *CYP3A4* rs28371759 (2.5%), *CYP4F2* rs2108622 (1.9%), *APOE* rs7412 (1.7%), and *VKORC1* rs2884737 (1.4%). In the dosing algorithm we developed, we confirmed the strongest effects of *VKORC1*, *CYP2C9* on warfarin dosing. In the limited sample set, we also found that novel genetic predictors (*CYP1A2*, *CYP3A4*, *APOE*, *EPHX1*, *CYP4F2*, and *VKORC1* rs2884737) may be associated with warfarin dosing. Further validation is needed to assess our results in larger independent northern Chinese samples.

## Abstract 2

Supplemental Digital Content is available in the text

## 1 Introduction

Valvular heart disease is one of the most common cardiovascular diseases, affecting over 100 million patients worldwide.^[1]^ Prosthetic (mechanical or bioprosthetic) heart valve replacement is an effective intervention for severe valvular heart disease, with approximately 30 million heart valves implanted every year globally.^[1]^ In China, more than 200,000 patients undergo prosthetic heart valve replacement surgery every year.^[2]^ Over 98% of these patients receive mechanical heart valve replacement (MHVR).^[3]^ MHVR patients must receive lifelong anticoagulation therapy for prevention of thrombosis. Warfarin is still the most frequently prescribed oral anticoagulant for MHVR patients.^[4]^ However, the effective dosage of warfarin is variable among patients, and incorrect dosage can lead to severe complications such as cerebral embolisms and life-threatening organ hemorrhages. Thus, it is important to be able to rapidly and precisely adjust warfarin dosage in the clinic.

Adjusting warfarin dosage is a challenging task, largely because there is a wide interindividual variability of warfarin dosing. Clinically, the warfarin dose can differ by up to 20-fold to achieve the same target international normalized ratio (INR). This is likely to be caused by differences in the genetic response to warfarin among patients. Studies in Caucasians and African-Americans show that *CYP2C9*,^[5]^*VKORC1*,^[6–8]^*CYP4F2*,^[9]^*EPHX1*,^[10]^*CALU*,^[11]^*APOE*,^[12,13]^ and *PROC*^[14]^ polymorphisms can significantly affect interindividual variability of warfarin dosing. In addition, several studies in Asian populations have found that polymorphisms in *GGCX*,^[15,16]^*NQO1*,^[17]^ and *HNF4α*^[18]^ are also associated with warfarin dosing, indicating that gene polymorphisms affecting warfarin dose requirements vary among different racial groups. The genetic makeup of the Chinese population differs from that of other ethnic groups; therefore, novel gene loci influencing warfarin dosing were found in Chinese patients. Using Sequenom's MassARRAY system, Lee et al^[19]^ found that *CYP2C18*, *PROC*, and *EPHX1* are associated with warfarin dosing in Taiwan Han Chinese patients, while Liang et al^[16]^ used the SNaPshot (Applied Biosystems, Foster City, CA, US) assay to identify *CYP2C19* rs3814637 and *GGCX* rs699664 as significantly associated with warfarin dosing in southwest Chinese patients.

These findings demonstrate that novel genetic variants affecting warfarin dosing should be identified in different ethnic groups to help determine precise warfarin therapy for a particular ethnic population. However, genetic effects on warfarin dosing vary among different Chinese ethnic groups, because people living in different regions of China differ in terms of history, lifestyle, culture, and dietary habits. However, most importantly, the studies mentioned above did not perform comprehensive genetic screens. Therefore, the present study recruited patients through a rigorous experimental design and genotyped 96 single nucleotide polymorphisms (SNPs) in 30 genes to identify the gene loci that may influencing warfarin dosing in northern Chinese MHVR patients. We then developed a new algorithm incorporating genetic and clinical factors to predict warfarin dose in this particular ethnic group.

## 2 Materials and methods

### 2.1 Patients

From April 2012 to May 2015, 226 ethnic Han Chinese patients who underwent MHVR were recruited from the 11th ward of the Cardiac Surgery Department of Anzhen Hospital, Capital Medical University, Beijing, China. The inclusion criteria were that patients had to be self-reported Han Chinese who lived in northern China (Beijing, Shandong, Shanxi, Henan, Hebei, Heilongjiang, Jilin, Liaoning, and Inner Mongolia Provinces), aged at least 18 years, able to provide written consent, administered warfarin for at least 3 months, and reached a stable maintenance dose. The stable maintenance dose was defined as a constant dose for at least 1 month within the target range of the INR. The target ranges were 1.6 to 2.0 for aortic valve replacement, 1.8 to 2.5 for mitral valve replacement, and 1.8 to 2.5 for a double valve replacement. This relatively low target INR was chosen because patients in Asian countries are more sensitive to warfarin therapy and are more likely to have bleeding events than Caucasians.^[20]^ Thus, most Chinese patients were routinely administrated low-intensity warfarin anticoagulation (target INR lower than 2–3), which has proved to be as effective as standard-intensity anticoagulation (target INR of 2–3) for preventing thromboembolic events, but is less likely to cause bleeding events.^[21–24]^ Exclusion criteria included hematological disease or hemorrhagic tendencies, blood platelet count <120 × 10^9^/L, heart failure (New York Heart Association class 3 or class 4), liver dysfunction (defined as the presence of chronic hepatic disease or biochemical evidence of significant hepatic impairment), kidney dysfunction (defined as the presence of chronic dialysis or renal transplantation or serum creatinine >200 mM), thyroid disease, malignant tumors, peptic ulcers, infections, autoimmune disease, pregnancy, and surgery or biopsy within the past 3 months.

Our study was carried out in accordance with the Declaration of Helsinki Principles (revised in 1983). All subjects provided written informed consent to participate in the study, which was reviewed and approved by the Ethics Committee of Beijing Anzhen Hospital, Capital Medical University, Beijing, China.

### 2.2 Clinical data collection

We collected patient data through regular telephone conversations, face-to-face interviews, and a review of medical records from our hospital information system. These data included age, sex, age at the time of operation, height, weight, the indication for warfarin treatment, position of valve prosthesis, valve types, routine INR values, stable warfarin dose, the concomitant disease, concurrent interacting medications, current smoking status, and alcohol consumption. The body surface area (BSA) was calculated as 0.0061 × height (cm) + 0.0128 × weight (kg) − 0.1529^[25]^; body mass index (BMI) was calculated as weight (kg)/(height [m^2^]). Hypertension was diagnosed after taking a mean of 3 independent measures of blood pressure >140/90 mm Hg; diabetes mellitus was diagnosed if the patient had a fasting glucose level >7.0 mmol/L, or a glucose level >11.1 mmol/L at 2 hours after oral glucose challenge, or both; hyperlipidemia was diagnosed with an elevation of at least one of the following: >6.22 mmol/L for total cholesterol, >2.26 mmol/L for triglycerides, or >4.14 mmol/L for low density lipoprotein-cholesterol. Smoking status was defined as self-reported use of cigarettes every day and smoked in the 30 days before follow-up; alcoholism was defined as an average daily consumption of 15 g or more pure alcohol in females and 25 g or more in males during the past 1 year.

### 2.3 DNA extraction

Eligible patients were recruited 1 day before surgery, and blood was collected for genotyping. Five milliliters of venous blood was collected from each patient and put into ethylenediaminetetraacetic acid vacuum tubes and stored at 4 °C before deoxyribonucleic Acid (DNA) extraction. Genomic DNA was extracted according to the instructions of the CWBIO Blood DNA System DNA isolation kit (CWBIO, Beijing, China) and stored at 4 °C for immediate use. DNA quality and purity were assessed by agarose gel electrophoresis, and optical absorbance was measured at A260/A280.

### 2.4 SNP selection

The polymorphisms analyzed in the present study included 96 SNPs within 30 genes (*VKORC1*, *GGCX*, *CALU*, *EPHX1*, *PROC*, *ORM1*, *F2*, *F5*, *F7*, *F9*, *APOE*, *NR1I2*, *STX4*, *NQO1*, *CACNA1C*, *FGFBP2*, *CYP2C9*, *CY92C8*, *CYP2C19*, *CYP2C18*, *CYP3A4*, *CYP1A1*, *CYP1A2*, *CYP4F2*, *CYP2A6*, *ABCB1*, *PROS1*, *CYP3A5*, *POR*, and *MGP*) (refer to table 1, Supplemental Content, which illustrates 30 candidate genes involved in the warfarin pharmacological pathway), which are all involved in transport, metabolism, or clearance of warfarin. The 96 candidate SNPs were selected from PharmGkb (https://www.pharmgkb.org) and have previously been reported to be involved in warfarin response in other ethnic groups. Search criteria included give preference to the genetic variants that have a higher “levels of evidence” (Level 1A > 1B > 2A > 2B > 3>  4) in PharmGkb Database; give preference to the variants that the “OMB Race Category” was “Mixed Population” or “Asian”; the variants which were reported in other races (“OMB Race Category” was “White,” “Black,” “African American,” or “Hispanic or Latino”) with more than 2 related publications. Those SNPs that the “OMB Race Category” was “White,” “Black,” “African American,” or “Hispanic or Latino,” with less than 2 related publications that support the evidence, and were not associated with warfarin dosage in reported publications were excluded.

### 2.5 SNP beadchip assay

Genotyping was performed using the Illumina SNP GoldenGate Assay (Illumina, San Diego, CA) according to the manufacturer's specifications. Briefly, 250 ng of genomic DNA was amplified at 37°C for 20 hours, and then the amplified DNA was fragmented and precipitated. The dried pellet was resuspended and hybridized to the beadchip. Hybridized beadchips were then incubated at 48 °C for 20 hours, washed, and a single-base extension step performed. After that, beadchips were stained, washed, coated, and dried. Finally, signal-intensity data were generated by an Illumina BeadArray Reader. We randomly selected 20% of the samples and genotyped them in duplicate, and 99.8% concordance was observed. The inconsistent data were excluded from the final analysis.

### 2.6 Statistical analysis

Descriptive statistics for the patients included means ± standard deviation (SD) or frequencies (n = 183). For all genetic polymorphisms, chi-square tests (or Fisher exact tests) were performed to ensure that the allele frequencies were within Hardy–Weinberg equilibrium. Warfarin dosage was square root–transformed to normalize distribution. Differences in the square root of warfarin stable dose between various genotype groups were analyzed by one-way analysis of variance (ANOVA) with post hoc comparison using least significant difference (LSD) analysis. Haploview (Broad Institute, Cambridge, MA, US) software was used to evaluate whether linkage disequilibrium existed between any of the genes.^[26]^ The variables of the variant allele were coded as 0, 1, and 2 for 0 copy, 1 copy, and 2 copies of the variant allele, respectively. Univariate association between the square root of warfarin maintenance dose and genetic polymorphisms was assessed using linear regression analysis. Univariate regression analysis was also performed using all clinical factors available, including age, gender, BSA, BMI, drinking and smoking habits, atrial fibrillation, hypertension, coronary artery disease, hyperlipidemia, diabetes mellitus, stroke history, valve positions, valve types, and administration of aspirin, β-blocker, amiodarone, statin, digoxin, ACE inhibitors, angiotensin II receptor antagonist, calcium channel blockers, insulin, diuretics, and isosorbide mononitrate. The candidate clinical factors with univariate analysis *P* < 0.15 (age, gender, BSA, concomitant atrial fibrillation, and coronary artery disease), together with 16 candidate genetic factors (*VKORC1* rs9923231, rs2884737, rs7196161, *STX4* rs11150606, *APOE* rs7412, *CYP2C9* rs1057910 (∗3), *CYP4F2* rs2189784 and rs2108622, *CYP1A2* rs2069514, *CYP3A4* rs28371759 and rs4686910, *EPHX1* rs2260863, *CALU* rs339097 and rs2290227, *PROC* rs2069910, and *ABCB1* rs1045642) were entered into stepwise regression analysis to develop a novel algorithm. Finally, Pearson correlation was performed to evaluate the correlation between observed and predicted doses. A 2-tailed probability value of <0.05 was considered significant. All statistical analyses were carried out using Statistical Package for Social Science (SPSS ver.20.0, SPSS Science, Chicago, IL).

## 3 Results

### 3.1 Patient characteristics

**Figure 1:**
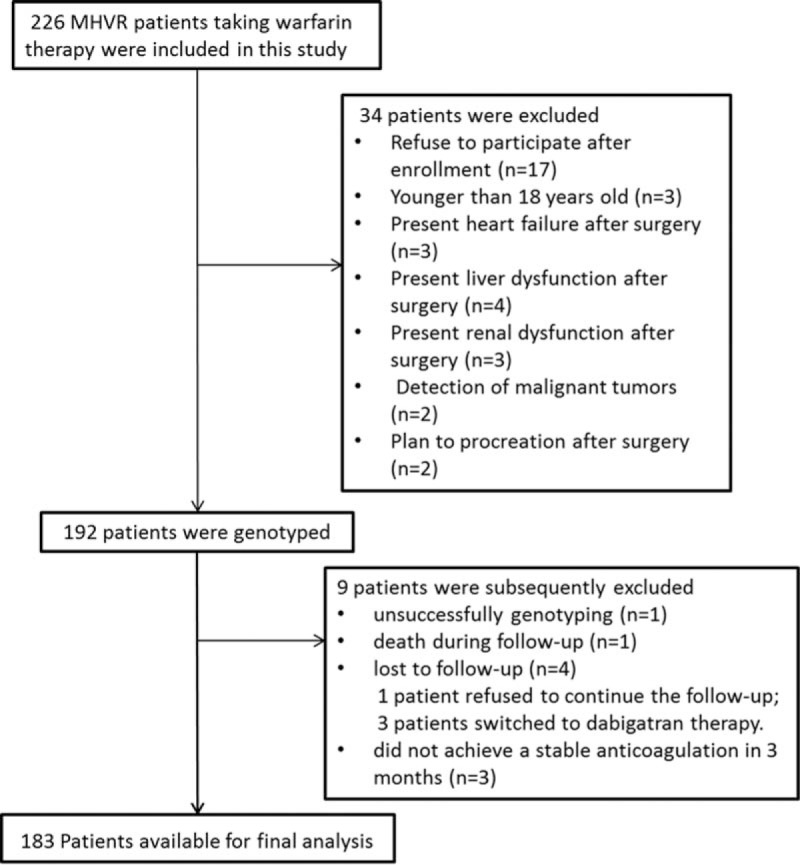
Flowchart of study enrollment and patient exclusion.

A total of 226 MHVR patients were enrolled in the study. Of these, 17 refused to participate after enrollment. Seventeen patients who did not meet the criteria were also excluded. Blood samples from 192 patients were collected for genotyping. One patient who was unsuccessfully genotyped was excluded from the study. The remaining 191 patients were followed up after surgery until August 2015. The follow-up period ranged from 4 to 35 months. During follow-up, 1 patient died of unexplained heart failure. Four patients were lost to follow-up because 1 patient refused to continue the follow-up and 3 patients switched to dabigatran therapy. Another 4 patients did not achieve stable anticoagulation over 3 months (Fig. 1). Finally, 183 patients with complete clinical and genetic data were included in the analysis. The baseline characteristics of these patients are listed in Table 1. The mean stable warfarin dose requirement was 3.10 ± 0.97 mg/d and ranged from 0.75 to 7.5 mg/d. Eleven patients experienced hemorrhages in the follow-up. Clinical factors with univariate *P* value <0.15 were shown in Table 2.

**Table 1:**
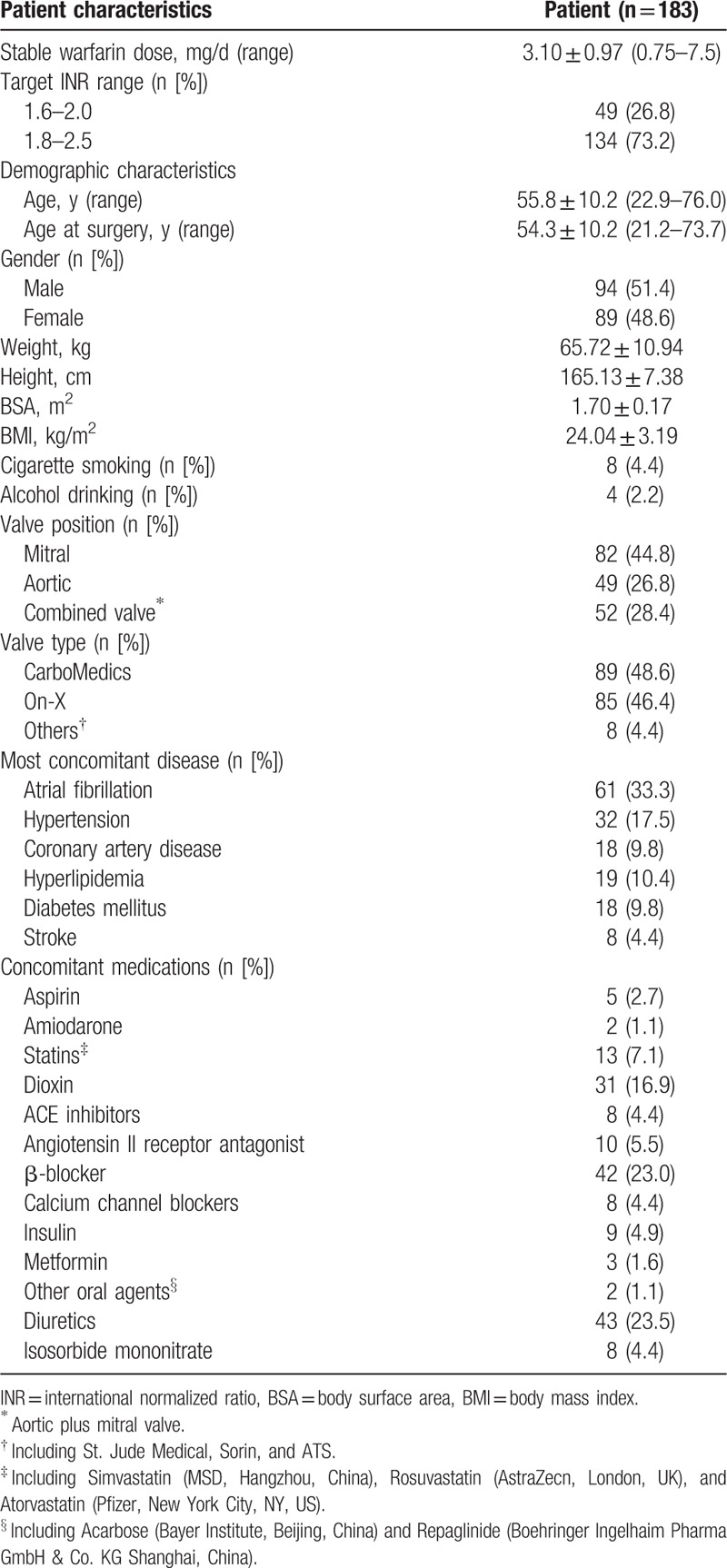
Demographic and clinical characteristics of MHVR patients.

**Table 2:**
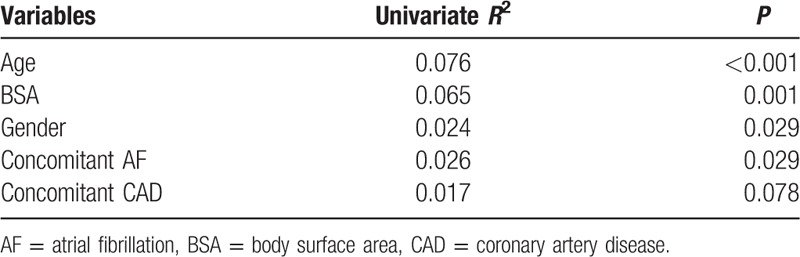
Candidate clinical variables with univariate regression *P* value <0.15.

### 3.2 Genes associated with warfarin maintenance dose

We selected 96 SNPs within 30 candidate genes that are involved in warfarin interactive pathways (Supplemental table 2, which illustrates the allele frequencies and genotype distribution of these SNPs). We searched the 1000 Genomes Browser (https://www.ncbi.nlm.nih.gov/variation/tools/1000genomes) and compared the MAF of all studied SNPs to the frequencies in 1000 genomes Han Chinese from Beijing (CHB). We found our data had the analogous frequencies compared to those data reported in 1000 genomes CHB (Supplemental table 2). Of these, 14 SNPs with a minor allele frequency of less than 1% were excluded from the subsequent analysis (including *CYP2C9∗2*). *VKORC1* rs17880887, *GGCX* rs669664, *CALU* rs1043550, and *MGP* rs1800801 deviated from the Hardy–Weinberg equilibrium and were also excluded from subsequent analysis. The remaining 78 SNPs were tested for association with warfarin dose. Univariate regression analysis showed that *VKORC1* rs9923231, *CYP2C9* rs1057910 (∗3), *CYP1A2* rs2069514, *CYP3A4* rs28371759, *EPHX1* rs2260863, *APOE* rs7412, and *CYP4F2* rs2189784 were significantly associated with a stable warfarin dose (all *P* < 0.05). In addition, *VKORC1* rs2884737, rs7196161, *STX4* rs11150606, *CALU* rs339097 and rs2290227, *PROC* rs2069910, *CYP3A4* rs4686910, *CYP4F2* rs2108622, and *ABCB1* rs1045642 showed univariate regression analysis with *P* < 0.20 (Table 3).

**Table 3:**
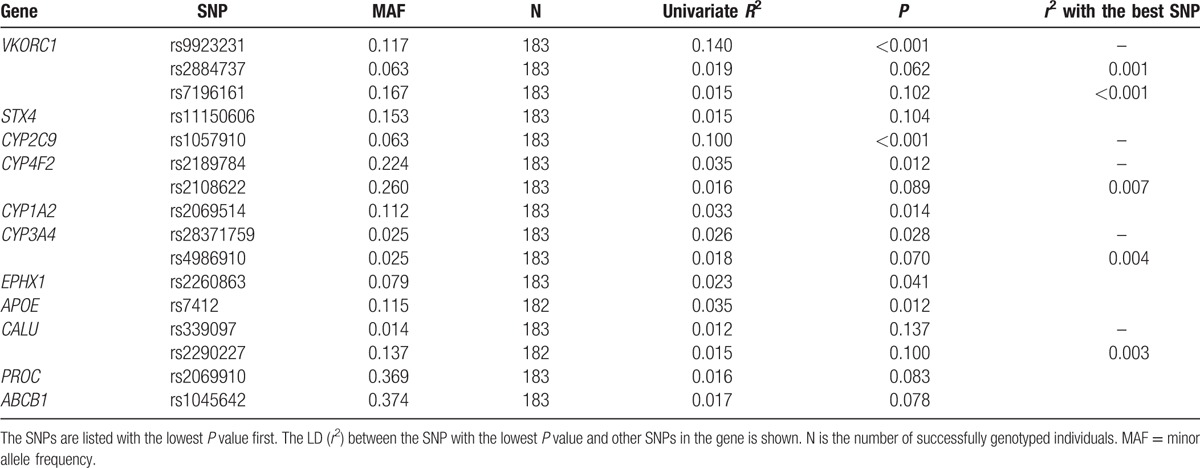
Association of candidate SNPs with stable warfarin dose, based on univariate regression of square root of dose.

**Figure 2:**
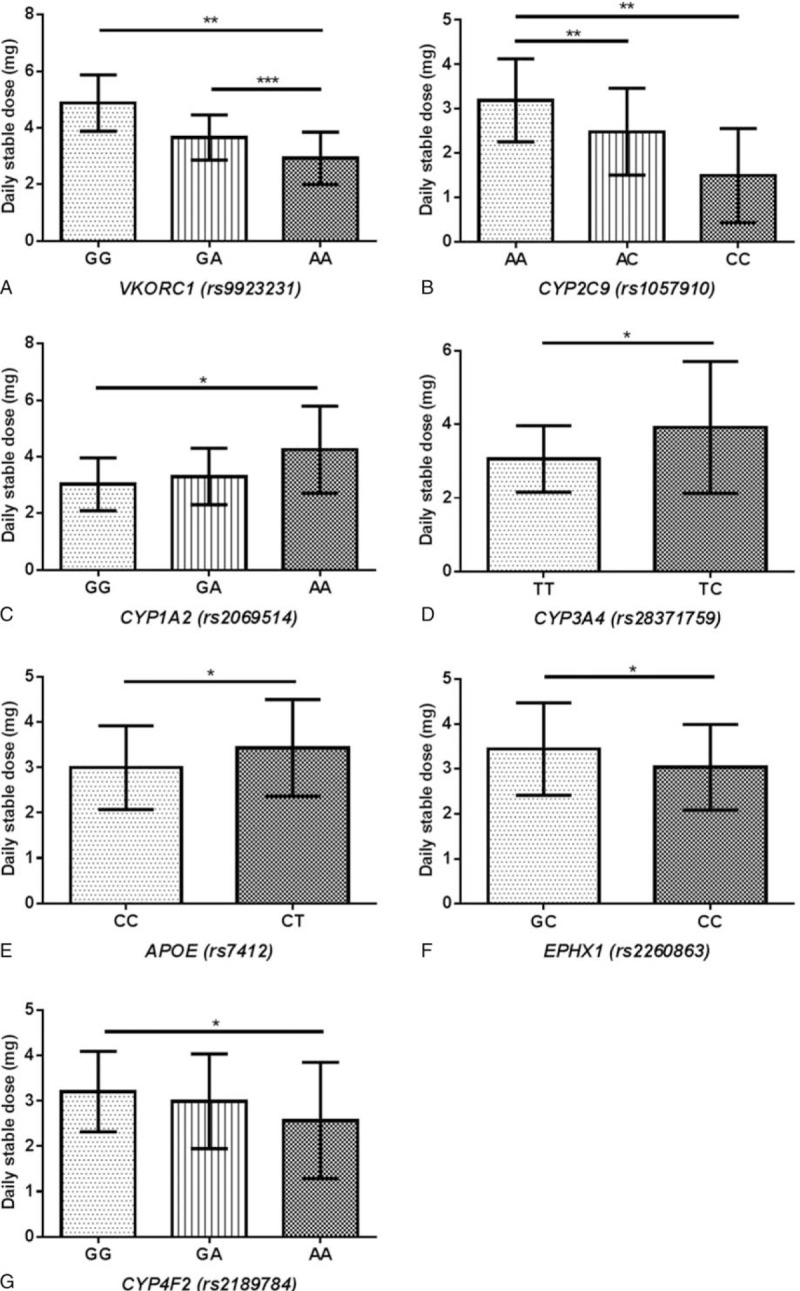
Bar diagram describing the relationships between genetic polymorphisms and daily stable warfarin doses (mg/d) in the study population (n = 183). Data are expressed as mean ± SD. (A) *VKORC1* rs9923231 polymorphisms. (B) *CYP2C9* rs1057910 (∗3) polymorphisms. (C) *CYP1A2* rs2069514 polymorphisms. (D) *CYP3A4* rs28371759 polymorphisms. (E) *EPHX1* rs2260863 polymorphisms. (F) *APOE* rs7412 polymorphisms. (G) *CYP4F2* rs2189784 polymorphisms. ^∗^Represents *P* < 0.05, ^∗∗^represents *P* < 0.01, ^∗∗∗^represents *P* < 0.001 (analyzed by one-way ANOVA with post hoc comparison using least significant difference analysis).

In the post hoc pairwise comparison, warfarin dose requirements were significantly higher in patients with *VKORC1* rs9923231 GG genotype compared with those with AA genotype (4.88 ± 0.99 vs 2.92 ± 0.93 mg/d, *P* < 0.01), and higher in GA genotype compared with AA genotype (3.66 ± 0.80 vs 2.92 ± 0.93 mg/d, *P* < 0.001) (Fig. 2A). Warfarin dose requirements were significantly higher in patients with *CYP2C9* rs1057910 1∗/1∗ genotype compared with 1∗/3∗ genotype (3.19 ± 0.93 vs 2.49 ± 0.98 mg/d, *P* < 0.01), and higher in 1∗/1∗ genotype compared with 3∗/3∗ genotype (3.19 ± 0.93 vs 1.50 ± 1.06 mg/d, *P* < 0.01) (Fig. 2B). For *CYP1A2* rs2069514, AA carriers required significantly higher warfarin dose than GG carriers (4.25 ± 1.54 vs 3.03 ± 0.93 mg/d, *P* < 0.05) (Fig. 2C). For *CYP3A4* rs28371759, TC heterozygous carriers required significantly higher dose than TT wild-type (3.92 ± 1.79 vs 3.06 ± 0.90 mg/d, *P* < 0.05) (Fig. 2D). Furthermore, individuals who carried an *APOE* rs7412 CT heterozygous genotype required a higher average dose than CC wild-type (3.44 ± 1.07 vs 3.00 ± 0.92 mg/d, *P* < 0.05) (Fig. 2E). Individuals who carried an *EPHX1* GC heterozygous genotype required significantly higher dose than CC wild-type (3.44 ± 1.03 vs 3.04 ± 0.95 mg/d, *P* < 0.05) (Fig. 2F). Last, *CYP4F2* GG carriers required significantly higher warfarin dose than AA mutant homozygotes (3.21 ± 0.89 vs 2.57 ± 1.28 mg/d, *P* < 0.05) (Fig. 2G) (relationships between other gene variants with univariate *P* value <0.2 figure 1, Supplemental Content).

### 3.3 Warfarin dosing algorithm

We analyzed the effect of nongenetic and genetic indicators using a stepwise forward elimination procedure. The observed effect size (*r*^2^) in the present study was compared with previous studies reporting these SNPs, as shown in Table 4. This model can be used to explain 44.4% of the variance in the stable warfarin dose. In this regression model, *VKORC1* and *CYP2C9* contributed most to the interindividual variability in warfarin dose, accounting for 14.2% and 9.6%, respectively. *CYP1A2* and *CYP3A4* contributed less than the above and increased the *r*^2^ by approximately 6.2%. The addition of *CYP4F2*, *APOE*, and *VKORC1* rs2884737 increased the *r*^2^ by 1.9%, 1.7%, and 1.4%, respectively. Besides, the nongenetic impact factors, including BSA and age, together contributed 9.4% to the interindividual variability.

**Table 4:**
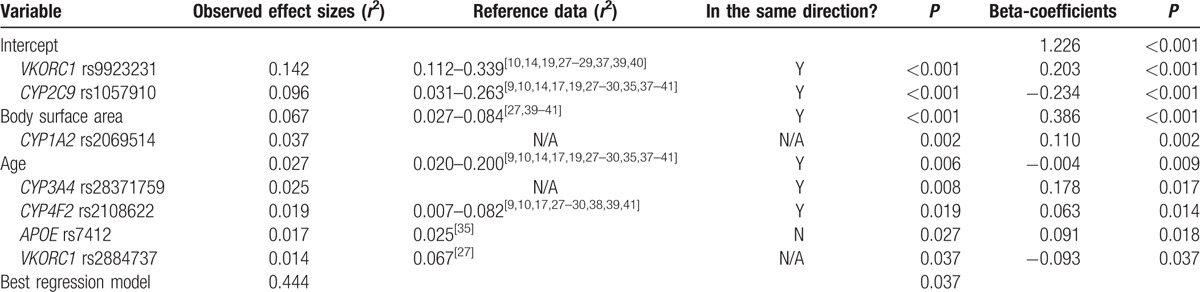
Final regression model produced by stepwise forward elimination procedure.

**Figure 3:**
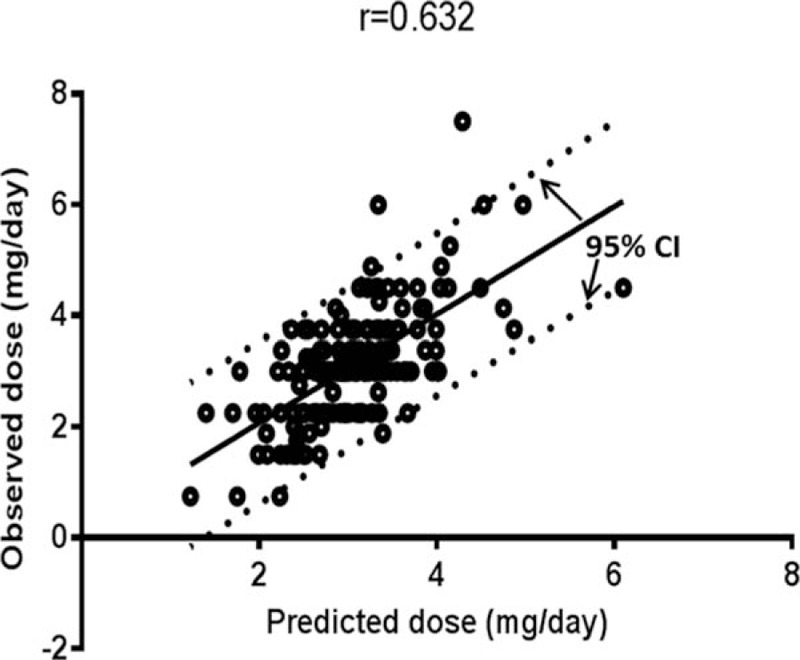
Relationship between dose predicted by the developed algorithm and observed dose in the study population (n = 183). Scatter plot of the predicted dose versus the observed dose. The solid line represents moderate correlation between actual and predicted dose (*r* = 0.632, *P* < 0.001). The dotted line represents a 95% confidence interval. (A value of *P* < 0.05 is considered significant, analyzed by Pearson correlation.) CI = confidence interval.

We then calculated the predicted dose using our multiple regression algorithm. Assessed by Pearson coefficient analysis, a moderately significant correlation between the predicted and observed warfarin maintenance doses was observed (Pearson *r* = 0.632, *P* < 0.001) (Fig. 3).

To derive a patient's maintenance dose using our algorithm, a clinician would apply the following equation using the patient's genetic and clinical data: square root of daily maintenance dose = 1.226 + 0.203 (*VKORC1* rs9923231) − 0.234 (*CYP2C9*∗3) + 0.386 (BSA) + 0.110 (*CYP1A2* rs2069514) − 0.004 (age) + 0.178 (*CYP3A4* rs28371759) + 0.063 (*CYP4F2* rs2108622) + 0.091 (*APOE* rs7412) − 0.093 (*VKORC1* rs2884737).

The coding of genetic variants was as follows: *VKORC1* rs9923231: input 0 for AA, 1 for AG, 2 for GG; *CYP2C9 ∗3*: input 1, 2, or 3 for the number of ∗3 alleles; *CYP1A2* rs2069514: input 0 for GG, 1 for GA, 2 for AA; *CYP3A4* rs28371759: input 0 for TT, 1 for TC, 2 for CC; *CYP4F2* rs2108622, input 0 for CC, 1 for CT, 2 for TT; *APOE* rs7412: input 0 for CC; 1 for CT; 2 for TT; *VKORC1* rs2884737, input 0 for AA, 1 for AC, 2 for CC.

## 4 Discussion

In the dosing algorithm we developed, we not only confirmed the strongest effects of VKORC1, CYP2C9, and CYP4F2 on warfarin dosing. In the limited sample set, we also found that novel genetic predictors (CYP1A2, CYP3A4, APOE, EPHX1, CYP4F2, and VKORC1 rs2884737) may be associated with warfarin dosing. Further validation is needed to assess our results in larger independent northern Chinese samples.

The present study is the largest screen performed to date to assess gene loci that are likely to affect warfarin dosing in the Chinese population. The results confirmed the strongest effects of *VKORC1*, *CYP2C9* on warfarin maintenance dose requirements. Moreover, the study demonstrated that *CYP1A2*, *CYP3A4*, *CYP4F2*, *APOE*, and *EPHX1* polymorphisms may be associated with warfarin dosing. By ANOVA analysis with post hoc comparison using LSD analysis, we found that *CYP1A2* rs2069514, *CYP3A4* rs28371759, and *APOE* rs7412 polymorphisms are associated with higher average warfarin dose, whereas *EPHX1* rs2260863 is associated with lower warfarin dose. By incorporating nongenetic factors (age and BSA), we developed a new algorithm which could explain 44.4% of the warfarin dosing variation in the northern Chinese patients. In the final model, *CYP1A2* rs2069514 and *CYP3A4* rs28371759 contributed 6.2% of interindividual variability of warfarin dosing, which to our knowledge, has not been previously reported.

Our results confirmed *VKORC1* and *CYP2C9* as major factors that influence interindividual variability of warfarin dosing. These 2 genetic factors together explain 23.8% of warfarin dose variation, which was similar to previous reports from other racial groups and people living in other regions of China. Our results also showed that *CYP4F2* rs2108622 contributed 1.9% of dose variation in the final algorithm. Extensive studies^[27–32]^ showed that the 3 genes mentioned above are the most common genetic factors that affect warfarin dose requirements in Caucasians, African-Americans, Hispanic-Americans, and Asians. In northern Chinese MHVR patients, these 3 predictors contribute to 24.7% of warfarin dose variation. Besides, in the present study, we found that *CYP4F2* rs2108622 was used for the final regression model instead of rs2189784, which showed smaller univariate *P* value than that of rs2108622. We speculated that it was due to the interactions between CYP4F2 and other gene expressions. Using STRING Database (http://string-db.org), we constructed the interactions between *CYP2C9* and *CYP4F2* gene expression products (data not shown). Subsequently, we removed CYP2C9 rs1057910 out of the multiple regression analysis. Interestingly, we found that CYP4F2 rs2189784 entered into the final model before rs2108622 (data not shown) when we removed genetic variable of CYP2C9 rs1057910. Further study may be conducted to valid this finding.

The present study found that genes encoding CYP family enzymes, which metabolize R-warfarin, were associated with warfarin maintenance dose. Three SNPs in *CYP1A2* were analyzed, and *CYP1A2* rs2069514, located in the 5′-flanking region, was significantly associated with warfarin dose requirements. *CYP1A2* rs2069514 AA carriers required significantly higher warfarin dose than GG carriers (4.25 ± 1.54 vs 3.03 ± 0.93 mg/d, *P* < 0.05). *CYP3A4*, which encodes an enzyme that metabolizes R-warfarin more potently, was associated with a higher warfarin dose. In the present study, we found that *CYP3A4* TC carriers required significantly higher dose than TT wild-type (3.92 ± 1.79 vs 3.06 ± 0.90 mg/d, *P* < 0.05). A recent study^[33]^ in Chinese patients with cyclosporine and tacrolimus therapy showed that *CYP3A4* rs28371759 variants are associated with increased CYP3A4 enzyme activity and increased substrate clearance. Similarly, our results show that carriers of the *CYP3A4* rs28371759C variant were associated with significantly higher warfarin dose requirements. This is because the C variant leads to increased enzyme activity and higher R-warfarin metabolism and clearance. *CYP1A2* and *CYP3A4* variants together contribute 6.2% of interindividual variability of warfarin dosing in the multiple regression model.

*APOE* and *EPHX1* variants were also associated with warfarin dose in the present study. Individuals who carried an *APOE* rs7412 CT heterozygous genotype required a higher average dose than CC wild-type (3.44 ± 1.07 vs 3.00 ± 0.92 mg/d, *P* < 0.05), similar to results from Huang et al^[34]^ in southwest Chinese patients. However, an Egyptian population shows contrasting results.^[35]^ Apolipoprotein E induces the uptake of vitamin K–rich lipoproteins by the liver. Changes in vitamin K content in the body can significantly affect warfarin anticoagulation activity and dose requirements. We postulate that *APOE* rs7412 variants have the slowest clearance of hepatic vitamin K leading to a reduction in the catabolism of vitamin K1, thus increasing the formation of active clotting factors in the circulating blood. Therefore, an increased warfarin dosage is required to achieve adequate anticoagulation effects. Furthermore, in the present study, we found that individuals who carried an *EPHX1* GC heterozygous genotype required significantly higher dose than CC wild-type (3.44 ± 1.03 vs 3.04 ± 0.95 mg/d, *P* < 0.05), but the effect was not observed in the multiple regression model. EPHX1 is proposed to be a putative subunit of the VKOR complex.^[36]^ The effect of EPHX1 may be confounded by the VKORC1 enzyme, because VKORC1 is the more important component of VKOR.

The performance of the pharmacogenetic algorithm in the present study was compared with the pharmacogenetic algorithm developed by the International Warfarin Pharmacogenetics Consortium (IWPC)^[37]^ in the low-dose (<2 mg/d), intermediate-dose (≥2–≤4 mg/d), and high-dose (>4 mg/d) groups (refer to Supplemental table 3, which illustrates the percentage of patients with underestimated, ideal, or overestimated dose of warfarin, as estimated by our pharmacogenetic algorithm and IWPC algorithms). Even though there was no significant difference observed between the 2 algorithms in patients who required a low dose (<2 mg/d) or a high dose (>4 mg/d), our algorithm considerably fare better than the IWPC algorithm in patients who required an intermediate dose (≥2–≤4 mg/d). These results indicate that the population-specific algorithm was more efficient than the multiethnic algorithm and is perhaps more suitable to a particular ethnic group. Recently, Chen et al^[38]^ developed an algorithm incorporating *VKORC1*, *CYP2C9*, *CYP4F2*, target INR, and clinical factors to predict warfarin dosing in a northern Chinese population. However, in our study we only recruited MHVR patients through our rigorous experimental design and screened a greater number of gene loci using the Illumina SNP GoldenGate Assay. We found that *CYP1A2*, *CYP3A4*, *APOE*, and *EPHX1* polymorphisms may also be associated with warfarin dosing variability. The present study was different from other algorithms in Taiwan Chinese^[18]^ and southwest Chinese populations,^[19]^ whose algorithms were based on other genetic predictors in the warfarin pharmacological pathway, such as polymorphisms of *CYP2C18*, *PROC*, *EPHX1*, *CYP2C19*, and *GGCX*. This is predictable because people living in the northern region of China differ in terms of history, lifestyle, culture, and dietary habits from people in other regions of China.

A limitation of our study is that our algorithm left some of the dose variability unaccounted for. For instance, some previously published algorithms included alcohol consumption, S-warfarin in the blood, or dietary vitamin K information. The contributions of these factors to the warfarin dose requirements were not assessed in the present study. Another limitation of this study was a relatively small sample size. Further validation is needed to assess our results in larger independent northern Chinese samples.

In conclusion, the present study recruited patients through a rigorous experimental design and screened 96 SNPs in 30 genes involved in the warfarin interactive pathway in northern Chinese MHVR patients. The results confirmed the strongest effects of *VKORC1* and *CYP2C9*. The results also found that *CYP1A2*, *CYP3A4*, *APOE*, *EPHX1*, *CYP4F2*, and *VKORC1* rs2884737 may be associated with warfarin dose requirements. Combined with clinical factors, we developed a multiple regression algorithm to explain 44.4% of warfarin dose variation in the northern Chinese population. Further validation is needed to assess our results in larger independent northern Chinese samples.

## Supplementary Material

Supplemental Digital Content
