# Supplementary material for: Clinical and genetic factors associated with warfarin maintenance dose in northern Chinese patients with mechanical heart valve replacement
Source: Medicine (Baltimore). 2017 Jan 13;96(2):e5658. doi: 10.1097/MD.0000000000005658 (PMC5266160; doi:10.1097/MD.0000000000005658)
Supplement: Supplemental Digital Content [file medi-96-e5658-s001.doc]

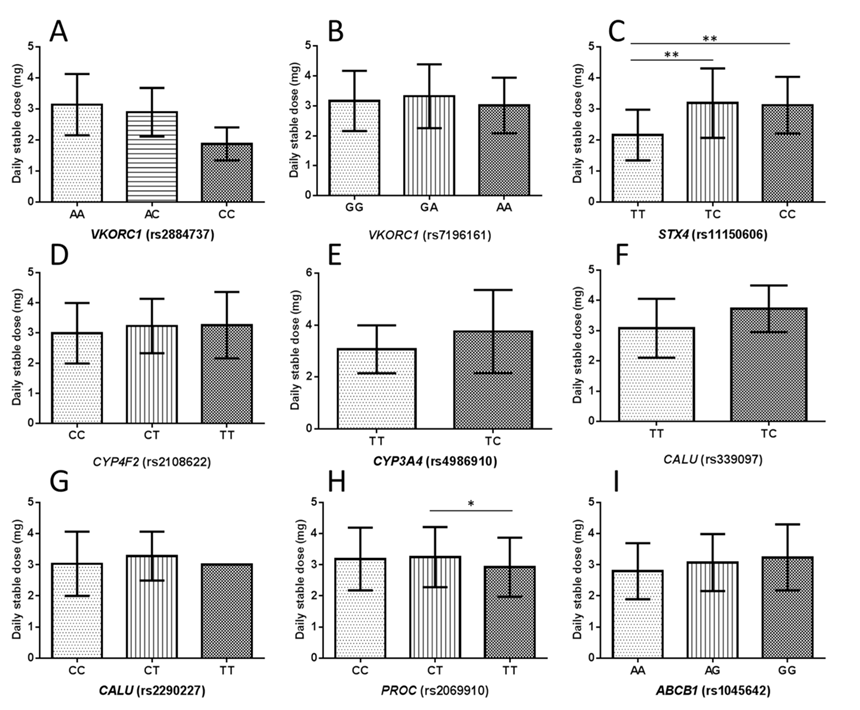


**Supplemental Fig. 2 Bar diagram describing the relationships between genetic polymorphisms and daily stable warfarin doses (mg/day) in the study population (n=183).** Data are expressed as mean ± SD. A) *VKORC1* rs2884737 polymorphisms. B) *VKORC1* rs7196161 polymorphisms. C) *STX4* rs11150606 polymorphisms. D) *CYP4F2* rs2108622 polymorphisms. E) *CYP3A4* rs4986910 polymorphisms. F) *CALU* rs339097 polymorphisms. G) *CALU* rs2290227 polymorphisms. H) *PROC* rs2069910 polymorphisms. I) *ABCB1* rs1045642 polymorphisms. * represents *P*<0.05, ** represents *P*<0.01, *** represents *P*<0.001, analysed by one-way ANOVA with post hoc comparison using LSD analysis.)

**Supplemental Table 1 30 candidate genes involved in the warfarin pharmacological pathway. Gene location is in NCBI build 144**.

| Gene symbol | Gene name | Location |
| --- | --- | --- |
| VKORC1 | Vitamin K epoxide reductase complex subunit 1 | Chr 16: 31090854-31096337bp |
| GGCX | Gamma-glutamyl carboxylase | Chr 2: 85544855-85561534bp |
| CALU | Calumenin | Chr 7:128739292-128773423bp |
| EPHX1 | Epoxide hydrolase 1, microsomal | Chr 1:225810074-225845563bp |
| PROC | Protein C | Chr 2:127418405-127429246bp |
| ORM1 | Orosomucoid 1 | Chr 9: 114323023-114326479bp |
| FII | Coagulation factor II | Chr 11:46719192-46739506bp |
| FV | Coagulation factor V | Chr 1:169511954-169586531bp |
| FVII | Coagulation factor VII | Chr 13:113105788-139563464bp |
| FIX | Coagulation factor IX | Chr X:139530734-139563464bp |
| APOE | Apolipoprotein E | Chr 19:44905749-44909395bp |
| NR1I2 | Pregnane X receptor | Chr 3:119780484-119818485bp |
| STX4 | Syntaxin 4 | Chr 16:31033095-31040168bp |
| NQO1 | NAD(P)H dehydrogenase, quinone 1 | Chr 16:69709401-69726668bp |
| CACNA1C | Calcium channel, voltage-dependent, L type, alpha 1C subunit | Chr 12:1970786-2697949bp |
| FGFBP2 | Fibroblast growth factor binding protein 2 | Chr 4:15960240-15963236bp |
| CYP2C9 | Cytochrome P_450_ 2C9 | Chr 10: 94938658-94989391bp |
| CYP2C8 | Cytochrome P_450_ 2C8 | Chr 10:95036772-95069498bp |
| CYP2C19 | Cytochrome P_450_ 2C19 | Chr 10:94762681-94853205bp |
| CYP2C18 | Cytochrome P_450_ 2C18 | Chr 10: 94683494-94736190bp |
| CYP3A4 | Cytochrome P_450_ 3A4 | Chr 7: 99756960-99784188bp |
| CYP1A1 | Cytochrome P_450_ 1A1 | Chr 15:74719542-74725610bp |
| CYP1A2 | Cytochrome P_450_ 1A2 | Chr 15:74748843-74756600bp |
| CYP4F2 | Cytochrome P_450_4F2 | Chr 19:15878024-15898120bp |
| CYP2A6 | Cytochrome P_450_2A6 | Chr 19:40843538-40850447bp |
| PROS1 | Protein S1 | Chr 3: 93873037-93974090bp |
| ABCB1 | P-glycoprotein | Chr7: 87503863-87713323bp |
| CYP3A5 | Cytochrome P_450_3A5 | Chr7: 99648189-99680026bp |
| POR | P450 (cytochrome) oxidoreductase | Chr7: 75915102-75986855bp |
| MGP | Matrix Gla protein | Chr 12:14881181-14885919bp |

**Supplemental Table 2 Allele frequencies and genotype distribution of candidate 96 SNPs in the analysed northern Chinese population**

| SNPs | Gene | Position | Allele | n | % | MAF | MAF  (CHB*) | Genotype | n | % | *P*-value HWE |
| --- | --- | --- | --- | --- | --- | --- | --- | --- | --- | --- | --- |
| rs9923231 | VKORC1 | 31096368 | A | 323 | 88.26 | 0.117 | 0.044 | AA | 143 | 78.14 | 0.7354 |
|  |  |  | G | 43 | 11.74 |  |  | AG | 37 | 20.22 |  |
|  |  |  |  |  |  |  |  | GG | 3 | 1.64 |  |
| rs7294 | VKORC1 | 31091000 | G | 345 | 94.26 | 0.057 | 0.044 | GG | 162 | 88.52 | 0.4103 |
|  |  |  | A | 21 | 5.74 |  |  | GA | 21 | 11.48 |  |
|  |  |  |  |  |  |  |  | AA | 0 | 0 |  |
| rs9934438 | VKORC1 | 31093557 | A | 318 | 86.89 | 0.131 | 0.044 | AA | 140 | 76.50 | 0.2295 |
|  |  |  | G | 48 | 13.11 |  |  | AG | 38 | 20.76 |  |
|  |  |  |  |  |  |  |  | GG | 5 | 2.73 |  |
| rs2359612 | VKORC1 | 31092475 | T | 342 | 93.42 | 0.066 | 0.044 | TT | 159 | 86.89 | 0.3425 |
|  |  |  | C | 24 | 6.58 |  |  | TC | 24 | 13.11 |  |
|  |  |  |  |  |  |  |  | CC | 0 | 0 |  |
| rs2884737 | VKORC1 | 31094233 | A | 343 | 93.72 | 0.063 | 0.005 | AA | 162 | 88.52 | 0.1089 |
|  |  |  | C | 23 | 6.28 |  |  | AC | 19 | 10.38 |  |
|  |  |  |  |  |  |  |  | CC | 2 | 1.09 |  |
| rs8050894 | VKORC1 | 31093188 | G | 342 | 93.44 | 0.066 | 0.044 | GG | 159 | 86.89 | 0.3425 |
|  |  |  | C | 24 | 6.56 |  |  | GC | 24 | 13.11 |  |
|  |  |  |  |  |  |  |  | CC | 0 | 0 |  |
| rs17708472 | VKORC1 | 31094032 | G | 366 | 100 | 0 | 0.000 | GG | 183 | 100 |  |
|  |  |  | A | 0 | 0 |  |  | GA | 0 | 0 |  |
|  |  |  |  |  |  |  |  | AA | 0 | 0 |  |
| rs61742245 | VKORC1 | 31094624 | A | 355 | 96.99 | 0.030 | 0.000 | AA | 172 | 93.99 | 0.6751 |
|  |  |  | C | 11 | 3.01 |  |  | AC | 11 | 6.01 |  |
|  |  |  |  |  |  |  |  | CC | 0 | 0 |  |
| rs17880887 | VKORC1 | 31099180 | C | 183 | 50.0 | 0.500 | 0.000 | CC | 0 | 0 | 1.07E-41 |
|  |  |  | A | 183 | 50.0 |  |  | CA | 183 | 100 |  |
|  |  |  |  |  |  |  |  | AA | 0 | 0 |  |
| rs17886199 | VKORC1 | 31093126 | T | 360 | 100 | 0 | 0.000 | TT | 180 | 100 |  |
|  |  |  |  | 0 | 0 |  |  |  | 0 | 0 |  |
|  |  |  |  |  |  |  |  |  | 0 | 0 |  |
| rs61162043 | VKORC1 | 31102913 | A | 0 | 0 | 0 | 0.044 | AA | 0 | 0 |  |
|  |  |  | G | 366 | 100 |  |  | AG | 0 | 0 |  |
|  |  |  |  |  |  |  |  | GG | 183 | 100 |  |
| rs104894539 | VKORC1 | 31094645 | G | 365 | 99.73 | 0.003 | 0.000 | GG | 182 | 99.45 | 0.9704 |
|  |  |  | T | 1 | 0.27 |  |  | GT | 1 | 0.55 |  |
|  |  |  |  |  |  |  |  | TT | 0 | 0 |  |
| rs104894540 | VKORC1 | 31094596 | T | 361 | 99.72 | 0.003 | 0.000 | TT | 180 | 99.45 | 0.9703 |
|  |  |  | C | 1 | 0.28 |  |  | TC | 1 | 0.55 |  |
|  |  |  |  |  |  |  |  | CC | 0 | 0 |  |
| rs104894541 | VKORC1 | 31094558 | A | 359 | 98.09 | 0.019 | 0.000 | AA | 176 | 96.17 | 0.7920 |
|  |  |  | G | 7 | 1.91 |  |  | AG | 7 | 3.83 |  |
|  |  |  |  |  |  |  |  | GG | 0 | 0 |  |
| rs104894542 | VKORC1 | 31091243 | T | 362 | 99.45 | 0.005 | 0.044 | TT | 180 | 98.90 | 0.9406 |
|  |  |  | G | 2 | 0.55 |  |  | TG | 2 | 1.10 |  |
|  |  |  |  |  |  |  |  | GG | 0 | 0 |  |
| rs11676382 | GGCX | 85550510 | C | 363 | 99.18 | 0.008 | 0.000 | CC | 180 | 98.36 | 0.9110 |
|  |  |  | T | 3 | 0.82 |  |  | CT | 3 | 1.64 |  |
|  |  |  |  |  |  |  |  | TT | 0 | 0 |  |
| rs669664 | GGCX | 85553413 | G | 281 | 76.78 | 0.232 | 0.058 | GG | 103 | 56.28 | 0.0435 |
|  |  |  | A | 85 | 23.22 |  |  | GA | 75 | 40.98 |  |
|  |  |  |  |  |  |  |  | AA | 5 | 2.73 |  |
| rs12714145 | GGCX | 85560218 | G | 237 | 64.75 | 0.352 | 0.354 | GG | 72 | 39.34 | 0.1252 |
|  |  |  | A | 129 | 35.25 |  |  | GA | 93 | 50.82 |  |
|  |  |  |  |  |  |  |  | AA | 18 | 9.84 |  |
| rs12777823 | GGCX | 94645745 | G | 218 | 59.56 | 0.404 | 0.340 | GG | 60 | 32.79 | 0.1308 |
|  |  |  | A | 148 | 40.44 |  |  | GA | 98 | 53.55 |  |
|  |  |  |  |  |  |  |  | AA | 25 | 13.66 |  |
| rs339097 | CALU | 128759170 | T | 361 | 98.6 | 0.014 | 0.010 | TT | 178 | 97.27 | 0.8514 |
|  |  |  | C | 5 | 1.4 |  |  | TC | 5 | 2.73 |  |
|  |  |  |  |  |  |  |  | CC | 0 |  |  |
| rs1043550 | CALU | 128769171 | A | 347 | 94.81 | 0.052 | 0.049 | AA | 166 | 90.71 | 0.0236 |
|  |  |  | G | 19 | 5.19 |  |  | AG | 15 | 8.20 |  |
|  |  |  |  |  |  |  |  | GG | 2 | 1.09 |  |
| rs11653 | CALU | 128769526 | T | 332 | 91.5 | 0.085 | 0.049 | TT | 150 | 82.42 | 0.1935 |
|  |  |  | A | 32 | 8.5 |  |  | TA | 32 | 17.58 |  |
|  |  |  |  |  |  |  |  | AA | 0 | 0 |  |
| rs2290228 | CALU | 128748594 | C | 280 | 76.50 | 0.235 | 0.218 | CC | 104 | 56.83 | 0.2018 |
|  |  |  | T | 86 | 23.50 |  |  | CT | 72 | 39.34 |  |
|  |  |  |  |  |  |  |  | TT | 7 | 3.83 |  |
| rs2290227 | CALU | 128748927 | C | 314 | 86.26 | 0.137 | 0.146 | CC | 133 | 73.08 | 0.1278 |
|  |  |  | T | 50 | 13.74 |  |  | CT | 48 | 26.37 |  |
|  |  |  |  |  |  |  |  | TT | 1 | 0.55 |  |
| rs1006023 | CALU | 128749500 | T | 344 | 94.51 | 0.055 | 0.049 | TT | 162 | 89.01 | 0.4328 |
|  |  |  | G | 20 | 5.49 |  |  | TG | 20 | 10.99 |  |
|  |  |  |  |  |  |  |  | GG | 0 | 0 |  |
| rs2307040 | CALU | 128754552 | G | 333 | 91.48 | 0.085 | 0.044 | GG | 151 | 82.97 | 0.2092 |
|  |  |  | A | 31 | 8.52 |  |  | AG | 31 | 17.03 |  |
|  |  |  |  |  |  |  |  | AA | 0 | 0 |  |
| rs339054 | CALU | 128742457 | A | 296 | 81.32 | 0.187 | 0.218 | AA | 117 | 64.29 | 0.1020 |
|  |  |  | C | 68 | 18.68 |  |  | AC | 62 | 34.07 |  |
|  |  |  |  |  |  |  |  | CC | 3 | 1.65 |  |
| rs1877724 | EPHX1 | 225825654 | C | 223 | 60.93 | 0.391 | 0.311 | CC | 66 | 36.07 | 0.5478 |
|  |  |  | T | 143 | 39.07 |  |  | CT | 91 | 49.73 |  |
|  |  |  |  |  |  |  |  | TT | 26 | 14.21 |  |
| rs4653436 | EPHX1 | 225807509 | G | 298 | 81.42 | 0.186 | 0.277 | GG | 120 | 65.57 | 0.5199 |
|  |  |  | A | 68 | 18.58 |  |  | GA | 58 | 31.69 |  |
|  |  |  |  |  |  |  |  | AA | 5 | 2.73 |  |
| rs2234922 | EPHX1 | 225838705 | A | 311 | 84.97 | 0.150 | 0.097 | AA | 135 | 73.77 | 0.0969 |
|  |  |  | G | 55 | 15.03 |  |  | AG | 41 | 22.40 |  |
|  |  |  |  |  |  |  |  | GG | 7 | 3.83 |  |
| rs2260863 | EPHX1 | 225832073 | C | 337 | 92.08 | 0.079 | 0.058 | CC | 154 | 84.15 | 0.2444 |
|  |  |  | G | 29 | 7.92 |  |  | CG | 29 | 15.85 |  |
|  |  |  |  |  |  |  |  | GG | 0 | 0 |  |
| rs1799809 | PROC | 127418299 | A | 263 | 71.86 | 0.281 | 0.175 | AA | 95 | 51.91 | 0.8530 |
|  |  |  | G | 103 | 28.14 |  |  | AG | 73 | 39.89 |  |
|  |  |  |  |  |  |  |  | GG | 15 | 8.20 |  |
| rs5936 | PROC | 127423296 | T | 212 | 58.24 | 0.418 | 0.490 | TT | 60 | 32.97 | 0.5967 |
|  |  |  | G | 152 | 41.76 |  |  | TG | 92 | 50.55 |  |
|  |  |  |  |  |  |  |  | GG | 30 | 16.48 |  |
| rs2069901 | PROC | 127417267 | T | 212 | 57.92 | 0.420 | 0.126 | TT | 56 | 30.60 | 0.1015 |
|  |  |  | C | 154 | 42.08 |  |  | TC | 100 | 54.64 |  |
|  |  |  |  |  |  |  |  | CC | 27 | 14.75 |  |
| rs2069910 | PROC | 127420398 | T | 231 | 63.11 | 0.369 | 0.320 | TT | 77 | 42.08 | 0.1927 |
|  |  |  | C | 135 | 36.89 |  |  | TC | 77 | 42.08 |  |
|  |  |  |  |  |  |  |  | CC | 29 | 15.85 |  |
| rs2069919 | PROC | 127421977 | G | 323 | 88.25 | 0.117 | 0.000 | GG | 141 | 77.05 | 0.2766 |
|  |  |  | A | 43 | 11.75 |  |  | GA | 41 | 22.40 |  |
|  |  |  |  |  |  |  |  | AA | 1 | 0.55 |  |
| rs1687390 | ORM1 | 114327608 | A | 281 | 76.78 | 0.232 | N/A | AA | 106 | 57.92 | 0.4381 |
|  |  |  | G | 85 | 23.22 |  |  | AG | 69 | 37.70 |  |
|  |  |  |  |  |  |  |  | GG | 8 | 4.37 |  |
| rs3817939 | F9 | 139530927 | A | 301 | 82.24 | 0.178 | 0.181 | AA | 127 | 69.40 | 0.1023 |
|  |  |  | G | 65 | 17.76 |  |  | AG | 47 | 25.68 |  |
|  |  |  |  |  |  |  |  | GG | 9 | 4.92 |  |
| rs510335 | F7 | 113105441 | G | 326 | 89.57 | 0.104 | 0.049 | GG | 144 | 79.12 | 0.1158 |
|  |  |  | T | 38 | 10.43 |  |  | GT | 38 | 20.88 |  |
|  |  |  |  |  |  |  |  | TT | 0 | 0 |  |
| rs510317 | F7 | 113105440 | G | 227 | 62.02 | 0.380 | 0.481 | GG | 66 | 36.07 | 0.1678 |
|  |  |  | A | 139 | 37.98 |  |  | GA | 95 | 51.91 |  |
|  |  |  |  |  |  |  |  | AA | 22 | 12.02 |  |
| rs5896 | F2 | 46723453 | T | 206 | 56.28 | 0.437 | 0.495 | TT | 53 | 28.96 | 0.1352 |
|  |  |  | C | 160 | 43.72 |  |  | TC | 100 | 54.64 |  |
|  |  |  |  |  |  |  |  | CC | 30 | 16.39 |  |
| rs6018 | F5 | 169542640 | T | 354 | 97.80 | 0.022 | 0.019 | TT | 173 | 95.58 | 0.7611 |
|  |  |  | G | 8 | 2.20 |  |  | TG | 8 | 4.42 |  |
|  |  |  |  |  |  |  |  | GG | 0 | 0 |  |
| rs429358 | APOE | 44908684 | T | 344 | 93.99 | 0.060 | 0.102 | TT | 161 | 87.98 | 0.3870 |
|  |  |  | C | 22 | 6.01 |  |  | TC | 22 | 12.02 |  |
|  |  |  |  |  |  |  |  | CC | 0 | 0 |  |
| rs7412 | APOE | 44908822 | C | 322 | 88.46 | 0.115 | 0.107 | CC | 140 | 76.92 | 0.0785 |
|  |  |  | T | 42 | 11.54 |  |  | CT | 42 | 23.08 |  |
|  |  |  |  |  |  |  |  | TT | 0 |  |  |
| rs2461818 | NR1I2 | 119805025 | T | 0 | 0 | 0 | 0.000 | TT | 0 | 0 |  |
|  |  |  | C | 366 | 100 |  |  | TC | 0 | 0 |  |
|  |  |  |  |  |  |  |  | CC | 183 | 100 |  |
| rs7643645 | NR1I2 | 119806650 | G | 195 | 53.28 | 0.467 | 0.452 | GG | 53 | 28.96 | 0.7544 |
|  |  |  | A | 171 | 46.72 |  |  | GA | 89 | 48.63 |  |
|  |  |  |  |  |  |  |  | AA | 41 | 22.40 |  |
| rs10871454 | STX4 | 31036758 | T | 340 | 93.41 | 0.066 | 0.039 | TT | 158 | 86.81 | 0.3410 |
|  |  |  | C | 24 | 6.59 |  |  | TC | 24 | 13.19 |  |
|  |  |  |  |  |  |  |  | CC | 0 | 0 |  |
| rs11150606 | STX4 | 31087690 | C | 310 | 84.70 | 0.153 | 0.170 | CC | 134 | 73.22 | 0.1213 |
|  |  |  | T | 56 | 15.30 |  |  | CT | 42 | 22.95 |  |
|  |  |  |  |  |  |  |  | TT | 7 | 3.83 |  |
| rs1800566 | NQO1 | 69711242 | T | 185 | 50.82 | 0.492 | 0.500 | TT | 41 | 8.51 | 0.0746 |
|  |  |  | C | 179 | 49.18 |  |  | TC | 103 | 56.59 |  |
|  |  |  |  |  |  |  |  | CC | 38 | 20.88 |  |
| rs216013 | CACNA1C | 2620466 | A | 270 | 63.77 | 0.262 | 0.262 | AA | 95 | 51.91 | 0.0795 |
|  |  |  | G | 96 | 26.23 |  |  | AG | 80 | 43.72 |  |
|  |  |  |  |  |  |  |  | GG | 8 | 4.37 |  |
| rs2189784 | CYP4F2 | 15848390 | G | 284 | 77.60 | 0.224 | 0.282 | GG | 110 | 60.11 | 0.9370 |
|  |  |  | A | 82 | 22.40 |  |  | GA | 64 | 34.97 |  |
|  |  |  |  |  |  |  |  | AA | 9 | 4.92 |  |
| rs2286461 | FGFBP2 | 15962050 | G | 207 | 56.56 | 0.434 | 0.398 | GG | 58 | 31.69 | 0.8717 |
|  |  |  | A | 159 | 43.44 |  |  | GA | 91 | 49.73 |  |
|  |  |  |  |  |  |  |  | AA | 34 | 18.58 |  |
| rs1799853 | CYP2C9 | 94942290 | C | 183 | 100 | 0 | 0.000 | CC | 183 | 100 |  |
|  |  |  | T | 0 | 0 |  |  | CT | 0 | 0 |  |
|  |  |  |  |  |  |  |  | TT | 0 | 0 |  |
| rs1057910 | CYP2C9 | 94981296 | A | 343 | 93.72 | 0.063 | 0.039 | AA | 162 | 88.52 | 0.1089 |
|  |  |  | C | 23 | 6.28 |  |  | AC | 19 | 10.38 |  |
|  |  |  |  |  |  |  |  | CC | 2 | 1.09 |  |
| rs9332127 | CYP2C9 | 94947714 | G | 344 | 93.99 | 0.060 | 0.039 | GG | 161 | 87.98 | 0.3870 |
|  |  |  | C | 22 | 6.01 |  |  | GC | 22 | 12.02 |  |
|  |  |  |  |  |  |  |  | CC | 0 | 0 |  |
| rs56165452 | CYP2C9 | 94981297 | A | 361 | 99.18 | 0.008 | 0.039 | AA | 179 | 98.35 | 0.9107 |
|  |  |  | G | 3 | 0.82 |  |  | AG | 3 | 1.65 |  |
|  |  |  |  |  |  |  |  | GG | 0 | 0 |  |
| rs72558187 | CYP2C9 | 94941958 | T | 358 | 97.81 | 0.022 | 0.000 | TT | 175 | 95.63 | 0.7624 |
|  |  |  | C | 8 | 2.19 |  |  | TC | 8 | 4.37 |  |
|  |  |  |  |  |  |  |  | CC | 0 | 0 |  |
| rs570317 | CYP2C9 | 48783974 | C | 366 | 100 | 0 | 0.393 | CC | 183 | 100 |  |
|  |  |  | T | 0 | 0 |  |  | CT | 0 | 0 |  |
|  |  |  |  |  |  |  |  | TT | 0 | 0 |  |
| rs7900194 | CYP2C9 | 94942309 | G | 359 | 98.09 | 0.019 | 0.000 | GG | 176 | 96.17 | 0.7920 |
|  |  |  | A | 7 | 1.91 |  |  | GA | 7 | 3.83 |  |
|  |  |  |  |  |  |  |  | AA | 0 | 0 |  |
| rs4917639 | CYP2C9 | 94965778 | T | 323 | 88.25 | 0.118 | 0.078 | TT | 140 | 76.50 | 0.0717 |
|  |  |  | G | 43 | 11.75 |  |  | TG | 43 | 23.50 |  |
|  |  |  |  |  |  |  |  | GG | 0 | 0 |  |
| rs4918758 | CYP2C9 | 94937495 | T | 184 | 50.83 | 0.492 | 0.408 | TT | 50 | 27.62 | 0.3356 |
|  |  |  | C | 178 | 49.17 |  |  | TC | 84 | 46.41 |  |
|  |  |  |  |  |  |  |  | CC | 47 | 25.97 |  |
| rs28371686 | CYP2C9 | 94981301 | C | 366 | 100 | 0 | 0.000 | CC | 183 | 100 |  |
|  |  |  | G | 0 | 0 |  |  | CG | 0 | 0 |  |
|  |  |  |  |  |  |  |  | GG | 0 | 0 |  |
| rs28371685 | CYP2C9 | 94981224 | C | 346 | 94.54 | 0.055 | 0.000 | CC | 163 | 89.07 | 0.4342 |
|  |  |  | T | 20 | 5.46 |  |  | CT | 20 | 10.93 |  |
|  |  |  |  |  |  |  |  | TT | 0 | 0 |  |
| rs9332108 | CYP2C9 | 94940223 | T | 332 | 92.74 | 0.073 | 0.039 | TT | 153 | 85.47 | 0.2947 |
|  |  |  | C | 26 | 7.26 |  |  | TC | 26 | 14.53 |  |
|  |  |  |  |  |  |  |  | CC | 0 | 0 |  |
| rs9325473 | CYP2C9 | 94974825 | G | 333 | 91.48 | 0.085 | 0.039 | GG | 154 | 84.62 | 0.1100 |
|  |  |  | A | 31 | 8.52 |  |  | GA | 25 | 13.74 |  |
|  |  |  |  |  |  |  |  | AA | 3 | 1.65 |  |
| rs1057911 | CYP2C9 | 94988980 | A | 346 | 94.54 | 0.055 | 0.039 | AA | 163 | 89.07 | 0.4342 |
|  |  |  | T | 20 | 5.46 |  |  | AT | 20 | 10.93 |  |
|  |  |  |  |  |  |  |  | TT | 0 |  |  |
| rs9332096 | CYP2C9 | 94937118 | C | 356 | 97.27 | 0.027 | 0.039 | CC | 173 | 94.54 | 0.7040 |
|  |  |  | T | 10 | 2.73 |  |  | CT | 10 | 5.46 |  |
|  |  |  |  |  |  |  |  | TT | 0 | 0 |  |
| rs7089580 | CYP2C9 | 94945466 | A | 355 | 97.53 | 0.025 | 0.024 | AA | 173 | 95.05 | 0.7323 |
|  |  |  | T | 9 | 2.47 |  |  | AT | 9 | 4.95 |  |
|  |  |  |  |  |  |  |  | TT | 0 | 0 |  |
| rs7196161 | CYP2C9 | 31099660 | A | 305 | 83.33 | 0.167 | 0.044 | AA | 128 | 69.95 | 0.6256 |
|  |  |  | G | 61 | 16.67 |  |  | AG | 49 | 26.78 |  |
|  |  |  |  |  |  |  |  | GG | 6 | 3.28 |  |
| rs10509680 | CYP2C9 | 94974582 | C | 338 | 93.37 | 0.066 | 0.039 | CC | 157 | 86.74 | 0.3394 |
|  |  |  | A | 24 | 6.63 |  |  | CA | 24 | 13.26 |  |
|  |  |  |  |  |  |  |  | AA | 0 | 0 |  |
| rs11572080 | CYP2C8 | 95067273 | G | 360 | 99.45 | 0.006 | 0.000 | GG | 179 | 98.90 | 0.9404 |
|  |  |  | A | 2 | 0.55 |  |  | GA | 2 | 1.10 |  |
|  |  |  |  |  |  |  |  | AA | 0 | 0 |  |
| rs3814637 | CYP2C19 | 94761288 | C | 287 | 78.42 | 0.216 | 0.087 | CC | 116 | 63.39 | 0.1292 |
|  |  |  | T | 79 | 21.58 |  |  | CT | 55 | 30.05 |  |
|  |  |  |  |  |  |  |  | TT | 12 | 6.56 |  |
| rs17882687 | CYP2C19 | 94762760 | A | 348 | 95.08 | 0.049 | 0.000 | AA | 165 | 90.16 | 0.4841 |
|  |  |  | C | 18 | 4.92 |  |  | AC | 18 | 9.84 |  |
|  |  |  |  |  |  |  |  | CC | 0 | 0 |  |
| rs7896133 | CYP2C18 | 94704973 | G | 326 | 89.07 | 0.109 | 0.087 | GG | 145 | 79.23 | 0.8878 |
|  |  |  | A | 40 | 10.93 |  |  | GA | 36 | 19.67 |  |
|  |  |  |  |  |  |  |  | AA | 2 | 1.09 |  |
| rs2901783 | CYP2C18 | 94693342 | A | 184 | 50.27 | 0.497 | 0.422 | AA | 42 | 22.95 | 0.2087 |
|  |  |  | G | 182 | 49.73 |  |  | AG | 100 | 54.64 |  |
|  |  |  |  |  |  |  |  | GG | 41 | 22.40 |  |
| rs2860840 | CYP2C18 | 94735475 | C | 290 | 79.23 | 0.208 | 0.209 | CC | 114 | 62.30 | 0.6890 |
|  |  |  | T | 76 | 20.77 |  |  | CT | 62 | 33.88 |  |
|  |  |  |  |  |  |  |  | TT | 7 | 3.83 |  |
| rs2242480 | CYP3A4 | 99763843 | C | 276 | 75.41 | 0.246 | 0.248 | CC | 101 | 55.19 | 0.2217 |
|  |  |  | T | 90 | 24.59 |  |  | CT | 74 | 40.44 |  |
|  |  |  |  |  |  |  |  | TT | 8 | 4.37 |  |
| rs28371759 | CYP3A4 | 99764003 | T | 357 | 97.54 | 0.025 | 0.000 | TT | 174 | 95.08 | 0.7331 |
|  |  |  | C | 9 | 2.46 |  |  | TC | 9 | 4.92 |  |
|  |  |  |  |  |  |  |  | CC | 0 | 0 |  |
| rs4686910 | CYP3A4 | 187951564 | T | 357 | 97.54 | 0.025 | 0.320 | TT | 174 | 95.08 | 0.7331 |
|  |  |  | C | 9 | 2.46 |  |  | TC | 9 | 4.92 |  |
|  |  |  |  |  |  |  |  | CC | 0 | 0 |  |
| rs1048943 | CYP1A1 | 74720644 | A | 271 | 74.45 | 0.255 | 0.267 | AA | 103 | 56.59 | 0.4088 |
|  |  |  | G | 93 | 25.55 |  |  | AG | 65 | 35.71 |  |
|  |  |  |  |  |  |  |  | GG | 14 | 7.69 |  |
| rs2069514 | CYP1A2 | 74745879 | G | 325 | 88.80 | 0.112 | 0.277 | GG | 146 | 79.78 | 0.2055 |
|  |  |  | A | 41 | 11.20 |  |  | GA | 33 | 18.03 |  |
|  |  |  |  |  |  |  |  | AA | 4 | 2.19 |  |
| rs762551 | CYP1A2 | 74749576 | A | 284 | 78.02 | 0.220 | 0.364 | AA | 108 | 59.34 | 0.2276 |
|  |  |  | C | 80 | 21.98 |  |  | AC | 68 | 37.36 |  |
|  |  |  |  |  |  |  |  | CC | 6 | 3.30 |  |
| rs11636419 | CYP1A2 | 74755259 | A | 342 | 93.44 | 0.066 | 0.262 | AA | 161 | 87.98 | 0.1433 |
|  |  |  | G | 24 | 6.56 |  |  | AG | 20 | 10.93 |  |
|  |  |  |  |  |  |  |  | GG | 2 | 1.09 |  |
| rs2108622 | CYP4F2 | 15879621 | C | 271 | 74.04 | 0.260 | 0.218 | CC | 101 | 55.19 | 0.7964 |
|  |  |  | T | 95 | 25.96 |  |  | CT | 69 | 37.70 |  |
|  |  |  |  |  |  |  |  | TT | 13 | 7.10 |  |
| rs1045642 | ABCB1 | 87509329 | G | 229 | 62.57 | 0.374 | 0.379 | GG | 66 | 36.07 | 0.0750 |
|  |  |  | A | 137 | 37.43 |  |  | GA | 97 | 53.01 |  |
|  |  |  |  |  |  |  |  | AA | 20 | 10.93 |  |
| rs12460590 | CYP2A6 | 40875742 | A | 241 | 65.85 | 0.342 | 0.354 | AA | 76 | 41.53 | 0.2714 |
|  |  |  | C | 125 | 34.15 |  |  | AC | 89 | 48.63 |  |
|  |  |  |  |  |  |  |  | CC | 18 | 9.84 |  |
| rs60711313 | CYP2A6 | 40843869 | T | 360 | 98.36 | 0.016 | N/A | TT | 177 | 96.72 | 0.8216 |
|  |  |  | C | 6 | 1.64 |  |  | TC | 6 | 3.28 |  |
|  |  |  |  |  |  |  |  | CC | 0 | 0 |  |
| rs8178607 | PROS1 | 93935148 | C | 350 | 95.63 | 0.044 | 0.083 | CC | 167 | 91.26 | 0.5363 |
|  |  |  | T | 16 | 4.37 |  |  | CT | 16 | 8.74 |  |
|  |  |  |  |  |  |  |  | TT | 0 | 0 |  |
| rs6976017 | CYP3A5 | 99652376 | G | 362 | 98.90 | 0.011 | 0.019 | GG | 179 | 97.81 | 0.8812 |
|  |  |  | A | 4 | 1.10 |  |  | GA | 4 | 2.19 |  |
|  |  |  |  |  |  |  |  | AA | 0 | 0 |  |
| rs2868177 | POR | 75960585 | A | 218 | 59.56 | 0.404 | 0.442 | AA | 61 | 33.33 | 0.2285 |
|  |  |  | G | 148 | 40.44 |  |  | AG | 96 | 52.46 |  |
|  |  |  |  |  |  |  |  | GG | 26 | 14.21 |  |
| rs1057868 | POR | 75985688 | C | 227 | 62.71 | 0.373 | 0.340 | CC | 77 | 42.54 | 0.0640 |
|  |  |  | T | 135 | 37.29 |  |  | CT | 73 | 40.33 |  |
|  |  |  |  |  |  |  |  | TT | 31 | 17.13 |  |
| rs1057909 | CYP2C9 | 94981294 | A | 362 | 99.45 | 0.005 | N/A | AA | 180 | 98.90 | 0.9406 |
|  |  |  | G | 2 | 0.55 |  |  | AG | 2 | 1.10 |  |
|  |  |  |  |  |  |  |  | GG | 0 | 0 |  |
| rs2860905 | CYP2C9 | 94942538 | G | 321 | 87.70 | 0.123 | N/A | GG | 138 | 75.41 | 0.0579 |
|  |  |  | A | 45 | 12.30 |  |  | GA | 45 | 24.59 |  |
|  |  |  |  |  |  |  |  | AA | 0 | 0 |  |
| rs9332146 | CYP2C9 | 94962487 | G | 357 | 97.54 | 0.025 | N/A | GG | 174 | 95.08 | 0.7331 |
|  |  |  | A | 9 | 2.46 |  |  | GA | 9 | 4.92 |  |
|  |  |  |  |  |  |  |  | AA | 0 | 0 |  |
| rs72553971 | POR | 75544247  (GRCh37.p13) | C | 364 | 99.45 | 0.005 | 0.000 | CC | 181 | 98.91 | 0.9407 |
|  |  |  | A | 2 | 0.55 |  |  | CA | 2 | 1.09 |  |
|  |  |  |  |  |  |  |  | AA | 0 | 0 |  |
| rs1800801 | MGP | 14885854 | G | 310 | 84.70 | 0.153 | 0.092 | GG | 127 | 69.40 | 0.0145 |
|  |  |  | A | 56 | 15.30 |  |  | GA | 56 | 30.60 |  |
|  |  |  |  |  |  |  |  | AA | 0 | 0 |  |

* CHB represents 1000 genomes Han Chinese in Beijing, China.

**Supplementary Table 3 Percentage of patients with underestimated, ideal or overestimated dose of warfarin, as estimated by our pharmacogenetic algorithm and IWPC algorithmsa (n=182b)**

| **Observed dose** | **No. of patients** | **Patients grouped based on the dose prediction (%)** | | | ***P*-value^d^** |
| --- | --- | --- | --- | --- | --- |
|  |  | Underestimation^c^ | Ideal dose^c^ | Overestimation^c^ |  |
| **<2mg/day (low dose)** | 19 |  |  |  |  |
| **Our algorithm** |  | 0 | 10.53(2) | 89.47(17) | - |
| **IWPC algorithm** |  | 0 | 0(0) | 100(19) | 0.486 |
| **2-4mg/day (intermediate dose)** | 136 |  |  |  |  |
| **Our algorithm** |  | 11.03 (15) | 70.59(96) | 18.38(25) | - |
| **IWPC algorithm** |  | 3.68(5) | 54.41(74) | 41.91(57) | 0.006 |
| **>4mg/day (high dose)** | 27 |  |  |  |  |
| **Our algorithm** |  | 55.56(15) | 40.74(11) | 3.70(1) | - |
| **IWPC algorithm** |  | 29.63(8) | 59.26(16) | 11.11(3) | 0.174 |
| **Total** | 182 |  |  |  |  |
| **Our algorithm** |  | 16.48(30) | 59.89(109) | 23.63(43) | - |
| **IWPC algorithm** |  | 7.14(13) | 49.45(90) | 43.41(79) | 0.045 |

IWPC, International Warfarin Pharmacogenetics Consortium

1. IWPC algorithms includes age, height, weight, *VKORC1* 1639 G/A, *CYP2C9*2*, *CYP2C9*3* (absent in our patients), enzyme Inducer and amiodarone.
2. One patient lack of genotyping data of *APOE* rs7412 to derive a predicted dose in our algorithm was excluded from analysis.
3. The ideal dose was defined as a predicted dose that was within 20% of the actual stable maintenance dose of warfarin, underestimation was defined as a predicted dose that was at least 20% lower than the actual dose, and overestimation was defined as a predicted dose that was at least 20% higher than the actual dose.
4. *p*, Value obtained from the comparison of our algorithm vs. IWPC algorithm, analysed by Chi-square tests (or Fisher’s exact tests).
